# The Association of 25 Hydroxyvitamin D and Parathyroid Hormone with Metabolic Syndrome in Two Ethnic Groups in South Africa

**DOI:** 10.1371/journal.pone.0061282

**Published:** 2013-04-15

**Authors:** Jaya A. George, Shane A. Norris, Hendrik Emmanuel van Deventer, Nigel J. Crowther

**Affiliations:** 1 Department of Chemical Pathology, National Health Laboratory Service and University of the Witwatersrand, Parktown, Johannesburg, South Africa; 2 Medical Reasearch Council/University of the Witwatersrand Developmental Pathways to Health Research Unit, University of the Witwatersrand, Parktown, Johannesburg, South Africa; 3 Lancet Laboratories, Auckland Park, Johannesburg, South Africa; Institute of Infectious Diseases and Molecular Medicine, South Africa

## Abstract

**Introduction:**

Though inconsistent, a number of studies have shown an association between vitamin D (25(OH)D) status, parathyroid hormone (PTH) and the metabolic syndrome (Met S). These have largely been carried out in Caucasians or black subjects living in high income countries. There no data on the relationship of 25(OH)D and PTH status with Met S in populations resident in Africa. The aims of this study were to evaluate if there was an association of 25(OH)D or PTH with Met S in non-Caucasian populations in South Africa, and whether these molecules explained ethnic differences in the prevalence of Met S and its individual components.

**Methods:**

We measured anthropometry, serum 25(OH)D and PTH levels and the components of Met S, plus related metabolic variables, in 374 African and 350 Asian Indian healthy adults from the greater Johannesburg metropolitan area.

**Results:**

Met S was diagnosed in 29% of the African and 46% of the Asian Indian subjects (p<0.0001). Subjects with Met S had higher PTH than those without Met S, (p<0.0001), whilst 25(OH)D levels were not significantly different (p = 0.50). In multivariate analysis, 25(OH)D was not associated with any components of the Met S however PTH was shown to be positively associated with systolic (p = 0.018) and diastolic (p = 0.005) blood pressures and waist circumference (p<0.0001) and negatively associated with HOMA (p = 0.0008) levels. Logistic regression analysis showed that Asian Indian ethnicity (OR 2.24; 95% CIs 1.57, 3.18; p<0.0001) and raised PTH (OR 2.48; 95% CIs 1.01, 6.08; p = 0.04; adjusted for 25(OH)D) produced an increased risk of Met S but 25(OH)D did not (OR 1.25; 95% CI 0.67, 2.24; p = 0.48).

**Conclusions:**

Plasma PTH but not 25(OH)D is an independent predictor of the Met S in African and Asian Indians in South Africa.

## Introduction

The incidence of obesity is on the rise in many low and middle income countries (LMICs) [1] and South Africa is no exception, with a national survey showing that 60% of South African women were either overweight or obese [2]. Obesity is associated with an increased risk of cardiovascular disease (CVD) and additional risk factors include hyperglycaemia or type 2 diabetes, hypertriglyceridemia, low high-density lipoprotein cholesterol (HDL-C), and hypertension. These CVD risk factors are commonly found together in subjects with abdominal obesity and this grouping together of CVD risk factors has been termed the metabolic syndrome (Met S) [3].

The burden of disease related to CVD is expected to increase substantially in LMIC, such as South Africa, over the next decades and it is important to understand traditional and non-traditional risk factors that contribute to this disease burden. Some populations are more susceptible to CVD because of specific factors – e.g. familial hypercholesterolaemia in the South African Afrikaner population [4] or insulin resistance in the Indian population [5]. The prevalence of cardiovascular and diabetes risk factors is higher in Asian Indian than African population groups in South Africa despite greater obesity in the latter population [6].

It has been suggested from several studies in various parts of the world that vitamin D deficiency or insufficiency may increase risk of the Met S and its sequelae, type 2 diabetes and CVD [7], [8]. Vitamin D is a hormone precursor which before exerting its metabolic effects undergoes two successive hydroxylation steps. The first converts vitamin D to 25 hydroxyvitamin D (25(OH)D) and the second converts 25(OH)D to the main active hormonal form 1,25 dihydroxy vitamin D (1,25-(OH)_ 2_D). Hypovitaminosis D appears to exert its effects via reductions in intracellular calcium and through the effects of 1,25(OH)_2_D on the regulation of various target genes e.g. by lack of suppression of the renin gene leading to hypertension [9], and reduced insulin secretion through reduction in islet β –cell function [8].

Elevated parathyroid hormone (PTH) levels have also emerged as a potential risk factor for Met S [10], [11]. PTH has been shown to be inversely associated with insulin sensitivity [12] and directly with blood pressure [13], [14]. Both 25(OH)D and PTH are calciotrophic hormones and their metabolism is closely related such that a decrease in serum 25(OH)D leads to a rise in PTH [15]. Currently, it is unclear whether the association noted between hypovitaminosis D and components of the Met S is mediated by 25(OH)D or PTH or by both.

There are no studies from Africa that have investigated any associations between 25(OH)D, PTH and components of the Met S. Therefore, the aims of this study were to examine the association of 25(OH)D and PTH with Met S in African and Asian Indian population groups in South Africa and to determine whether ethnic differences in 25 (OH)D or PTH levels contribute to differences in the prevalence of Met S, its component parts and related metabolic variables, within these populations. The hypotheses to be tested were that 25(OH)D is lower and PTH is higher in Met S patients in both population groups and that ethnic differences in the prevalence of Met S and its component parts are related to ethnic differences in the blood level of 25(OH)D.

## Research Design and Methods

### Subjects

This was a cross sectional study of African and Asian Indians from the greater Johannesburg-Soweto metropolitan area in South Africa. Participants were recruited via the Birth to Twenty study, which is a longitudinal analysis of more than 3200 children and their caregivers which began in 1990 and is representative of long-standing urban inhabitants [16]. Caregivers of the cohort were contacted and asked if they had any family members or neighbours that would be interested in participating. Exclusion criteria consisted of pregnancy, breast feeding, renal failure, age below 18 or above 70, Caucasian or persons of mixed ancestry. A total of 730 participants were recruited, and were stratified by ethnicity, age and sex. Ethnicity was self-reported as African or Asian Indian. Ethics clearance was obtained from the Human Ethics Committee of the University of the Witwatersrand and written informed consent was obtained from each subject.

### Biochemistry

After an overnight fast of at least 8 hours, peripheral venous blood was collected in EDTA tubes for 25(OH)D, PTH and glycated haemoglobin analysis (HbA1c), in fluoride tubes for glucose and in plain tubes for the rest of the analytes. Tubes were kept on ice and centrifuged after 30 minutes at 3500 g for 10 minutes. Glucose, HbA1c and lipid profile were assayed on the day of collection and aliquots for 25(OH)D, PTH, and insulin were stored at −80°C until analysis. Glucose, total cholesterol, HDL-C and triglycerides were measured enzymatically on an automated analyser (Siemens ADVIA 1800, Tarrytown, USA). The HbA1c was measured on the ADVIA 1800, which was standardized according to the Diabetes Control and Complications Trial National Glycohemoglobin Standardization Program [17]. Creatinine was measured by a modified Jaffe test and fasting insulin and PTH were measured in batches by a chemiluminescence assay on the ADVIA Centaur (Siemens Diagnostics, Tarrytown, USA). The intra assay intra assay coefficient of variation (CV) for PTH ranges from 5.2% at 4.3 pmol/L to 3.4% at 23.7 pmol/L.

Plasma 25(OH)D was measured using the Clin Rep high performance liquid chromatography (HPLC) kit (Recipe, Munchen, Germany). In this analytical method, 25(OH)D was determined from plasma using HPLC with a photodiode array (PDA) detector. Prior to HPLC analysis a short liquid-liquid extraction was performed. Thereafter the samples were injected onto the HPLC system and the analytes were separated on the appropriate analytical column. The separated 25(OH)D_2_ and 25 (OH)D_3_ were detected at a wavelength of 264 nm. The chromatograms were integrated using peak height. Total vitamin D (25(OH)D) was taken as the sum of 25(OH)D_2_ and 25(OH)D_3_. The intra assay CV for 25(OH)D ranged from 0.9–4.9% and the inter-assay CV ranged from 3.0– 4.9%.The limit of detection was 2.5 nmol/l for 25(OH)D_3_ and 7.5 nmol/L for 25(OH)D_2_. Our laboratory participates in the vitamin D external quality assurance scheme (DEQAS).

### Diagnosis of Metabolic Syndrome, and Measurement of Insulin Resistance and Glomerular Filtration Rate

Met S was determined using the harmonized definition [3], [17]. Thus, subjects had to have at least three of the following criteria: elevated waist circumference (≥94 cm for African males, ≥80 cm for African or Asian Indian females, ≥90 cm for Asian Indian males); elevated serum triglycerides (≥1.7 mmol/L); reduced serum HDL-C (<1.0 mmol/L for males and 1.3 mmol/L for females); elevated blood pressure (systolic blood pressure ≥130 mmHg and/or diastolic pressure ≥85 mmHg) and elevated fasting blood glucose (≥5.6 mmol/L). Insulin resistance was measured using the homeostasis model assessment (HOMA) technique [18] and glomerular filtration rate (GFR) was calculated by a modified MDRD formula [19].

### Medical History and Anthropometric Data Collection

A standardized questionnaire was used to collect information on age, sex, visit date, smoking (current, former, never), self-reported diabetes, hypertension, dyslipidaemia, coronary heart disease and medication use. Body weight and height were measured with subjects in light clothing and without shoes using an electronic scale and wall mounted stadiometer (Holtain, UK). The waist circumference was measured with a soft measuring tape to the nearest 0.5 cm at the level of the smallest girth above the umbilicus in the standing position. Blood pressure was measured thrice in the right arm, with the subject seated and after 5 minutes rest using an automated sphygmomanometer. The final reading was used.

### Statistical Methods

Stata 12 (College Station, TX, USA) was used for all analyses. The distribution of variables was assessed by the Shapiro-Wilk’s W test and by examination of the normal probability plot. Logarithmic transformations were applied for all non-normally distributed variables. In descriptive analysis, normally distributed, continuous variables were reported as means ± SD whilst non-normally distributed data were expressed as median (interquartile range). Categorical data were reported as N (percent).

Pearson’s correlation was used to assess the association of 25(OH)D and PTH with the selected variables. Multivariate regression analyses were conducted to assess the association of the Met S components with 25(OH)D and PTH after adjusting for potential confounders i.e. age, gender and body mass index (BMI). Ethnicity was also included as an independent variable to determine if ethnic differences in Met S components were mediated by 25(OH)D or PTH.

In order to determine if the higher risk of Met S in the Indian population was mediated by 25(OH)D or PTH, logistic regression analysis was performed with the stepwise addition of 25(OH)D and PTH to a model that also included ethnicity, age, gender and BMI as independent variables. The attenuation of the p-value (from significant to non-significant) for the ethnicity odds ratio was used as the indicator of any effect.

PTH was found to increase the risk of Met S and therefore we analysed which of the individual components of the Met S were influenced by PTH by separately adding each individual component to a logistic regression model for MetS risk which included PTH, age, gender, ethnicity, BMI and 25(OH)D as independent variables. As described above the attenuation of the p-value, in this case for the PTH odds ratio, was used as the effect indicator.

In all regression models Africans were coded as 0 and Asian Indians as 1whilst females were coded as 0 and males as 1.

## Results

Six participants were excluded from the analyses due to incomplete data collection and seven participants with serum calcium above the normal reference range (2.15–2.50 mmol/L) were also excluded. Characteristics of the participants (n = 717) are shown in Table 1. Both ethnic groups had similar (p>0.05) age, BMI, HDL-C, GFR, calcium and PTH levels. Asian Indian subjects had significantly higher fasting glucose, triglycerides and total cholesterol (p<0.0001 for each of the 3 comparisons) compared to the African population. They also had a greater waist circumference (p = 0.0001) and were more insulin resistant (p<0.0001). African subjects had higher 25(OH)D levels and systolic and diastolic blood pressures (p<0.0001 for all three comparisons). The prevalence of 25(OH)D deficiency (defined as 25(OH)D <30 nmol/L [20]) was 3.0% in the African and 15.0% in the Asian Indian population (p<0.0001). The month of the year during which blood samples were drawn for 25(OH)D measurement may be a confounding variable since 25(OH)D levels are known to vary across the seasons [21]. However, ethnic differences in plasma 25(OH)D levels were still observed irrespective of the season in which the blood sample was obtained. Vitamin D levels were highest for both ethnic groups during the autumn months (data not shown).

**Table 1:** Characteristics of subjects according to ethnicity and gender.

Variable	African	AsianIndian	p-values	AfricanMales	AfricanFemales	p-values	Asian Indian Males	Asian Indian Females	p-values
***N***	373	344	–	181	192	–	161	183	–
***Age (years)***	41.6±13.1	43.5±12.9	0.06	42.0±13.2	42.0±13.1	0.9	42.0±13.2	46.0±12.7	0.5
***Glucose (mmol/L)***	4.9 (4.6, 5.0)	5.21 (4.80, 5.70)	<0.0001	4.90 (4.60, 5.40)	4.90 (4.60, 5.30)	0.5	5.20 (4.91, 5.80)	5.10 (4.80, 5.63)	0.03
***Triglycerides*** ***(mmol/L)***	0.85 (0.62, 1.27)	1.26 (0.83, 1.85)	<0.0001	0.93 (0.67, 1.42)	0.82 (0.57, 1.17)	0.03	1.37 (1.00, 2.08)	1.10 (0.77, 1.60)	<0.0001
***HDL-C (mmol/L)***	1.28 (1.08, 1.53)	1.26 (1.07, 1.53)	0.81	1.20 (1.01, 1.43)	1.33 (1.17, 1.59)	<0.0001	1.12 (1.00, 1.26)	1.45 (1.24, 1.73)	<0.0001
***Systolic blood pressure (mmHg)***	131 (120, 146)	123 (113, 134)	<0.0001	133 (121, 148)	129 (119, 143)	0.16	125 (118, 135)	118 (108, 132)	0.0003
***Diastolic blood pressure (mmHg)***	83 (76, 93)	80.0 (73.1, 87.0)	<0.0001	82.0 (74.0, 93.0)	83.0 (77.3, 93.2)	0.40	82.0 (75.3, 89.1)	78.2 (72.0, 85.2)	0.006
***Waist (cm)***	89.0 (78.0, 102)	95.0 (85.0, 106)	0.0001	85.0 (75.0, 99.0)	95.0 (82.0, 105)	<0.0001	98.0 (90.5, 108)	93.0 (82.0, 103)	0.0006
***GFR (ml/min/1.73*** ***m^2^)***	88.0±22.4	88.1±19.7	0.9	88.7±28.7	87.4±22.5	0.92	84.2±17.4	92.9±20.4	<0.0001
***Calcium (mmol/L)***	2.27±0.09	2.26±0.11	0.21	2.28±0.08	2.27±0.10	0.32	2.26±0.1	2.26±0.1	0.63
***Cholesterol (mmol/L)***	4.22±1.04	4.97±1.10	<0.0001	3.93±0.99	4.24 1.07	0.002	4.72±1.17	4.97±1.04	0.16
***BMI (kg/m^2^)***	26.2 (21.7, 31.7)	26.7 (23.3, 31.0)	0.38	23.3 (20.2, 27.3)	29.9 (24.3, 35.3)	<0.0001	26.2 (23.7, 30.4)	27.1 (23.0, 32.8)	0.36
***HOMA***	1.83 (1.1, 2.9)	3.23 (2.13, 5.01)	<0.0001	1.43 (0.80, 2.69)	2.16 (1.31, 2.92)	0.0001	3.45 (2.15, 5.50)	3.15 (2.05, 4.60)	0.12
***PTH (pmol/L)***	4.70 (3.3, 6.5)	4.85 (3.61, 6.92)	0.09	4.30 (3.00, 5.60)	5.30 (3.80, 5.90)	0.0002	4.60 (3.60, 6.40)	5.00 (3.70, 7.20)	0.09
***25 (OH) D (nmol/L)***	70.9 (51.5, 95.1)	41.8 (28.6, 56.8)	<0.0001	72.7 (51.1, 94.1)	58.3 (42.9, 85.6)	0.0008	46.8 (33.6, 62.7)	35.7 (23.0, 54.5)	0.0002

Data given as mean ± SD for normally distributed data and median (IQR) for non-normally distributed data.

In both ethnic groups females had lower 25(OH)D levels compared to their male counterparts (p = 0.0008 in Africans and p = 0.0002 in Asian Indians) and this was also observed for triglyceride levels (p = 0.03 in Africans and p<0.0001 in Asian Indians) (Table 1). The PTH levels were higher in African females than males (p = 0.0002), and although the same pattern was observed in Asian Indians, the gender effect was not significant (p = 0.09). No gender differences were observed for age or calcium levels in either ethnic group. In African females, cholesterol (p = 0.002), BMI (p<0.0001) and HOMA (p = 0.0001) were higher than in males, but no gender effect for these variables was seen in the Indian population. Asian Indian females had higher GFR than Asian Indian males (p<0.0001) but no gender difference was observed in the African cohort. In Asian Indian males, glucose (p = 0.03) and systolic (p = 0.0003) and diastolic (p = 0.006) blood pressure were higher than in females but no differences were noted between sexes in the African subjects. Within both ethnic groups, HDL-C levels were higher in females than males (p<0.0001 for both). Waist circumference was significantly higher in African females than males (p<0.0001), but in the Asian Indian subjects this trend was reversed (p = 0.0006).

Met S was diagnosed in 38% of the participants, with a prevalence of 29% in the African and 46% in the Asian Indian group (p<0.0001). The data in Table 2 show that within each ethnic group the most prevalent Met S component was elevated waist circumference (94.5% in Africans and 96.9% in Asian Indians; p = 0.33) and elevated triglyceride was the least common (36.7% in Africans and 54.7% in Asian Indians; p = 0.004). The occurrence of raised fasting glucose levels was more common in Asian Indian than African Met S patients (p = 0.009), whilst raised systolic blood pressure was more common in African than Asian Indian Met S subjects (p = 0.005). Low HDL-C (p = 0.28) and raised diastolic blood pressure (p = 0.13) were not significantly different in frequency across the two ethnic groups.

**Table 2:** Prevalence of individual components of the Met S in each ethnic group.

Variable	Africans with Met S (N = 109)	Asian Indians with Met S (N = 161)	p-values
***Raised glucose***	48 (44.0)	97 (60.3)	0.009
***Raised triglycerides***	40 (36.7)	88 (54.7)	0.004
***Reduced HDL-C***	74 (67.9)	91 (61.5)	0.28
***Raised systolic blood pressure***	96 (88.9)	121 (75.2)	0.005
***Raised diastolic blood pressure***	83 (76.9)	110 (68.3)	0.13
***Raised waist circumference***	103 (94.5)	156 (96.9)	0.33

Data given as N (%).

In both ethnic groups, subjects with Met S had higher ages, HbA1c, HOMA, PTH, cholesterol and BMI measures than those without the Met S, whilst GFR was lower in those with Met S (p<0.05 for all comparisons) (see Table 3). Calcium (p = 0.80) and 25(OH)D levels (p = 0.50) were not significantly different across these 2 groups.

**Table 3:** Comparison of subjects with and without Met S within each ethnic group.

	Africans without Met S	Africans with Met S	p-values	Asian Indianswithout Met S	Asian Indians with Met S	p-values
***N***	260	109	–	186	158	–
***Age (years)***	39.1±12.9	47.7±11.3	<0.0001	38.4±12.7	49.4±10.5	<0.0001
***HbA1c (%)***	5.31 (5.06, 5.32)	5.64 (5.35, 6.36)	<0.0001	5.33 (5.04, 5.56)	5.76 (5.41, 6.13)	<0.0001
***HOMA***	1.44 (0.87, 2.35)	2.85 (2.00, 4.00)	<0.0001	2.43 (1.72, 3.58)	4.56 (2.99, 6.77)	<0.0001
***PTH (pmol/L)***	4.50 (3.25, 5.95)	5.41 (3.92, 7.60)	0.0002	4.40 (3.50, 6.20)	5.40 (4.13, 7.62)	0.0001
***25(OH)D (nmol/L)***	72.3 (52.6, 95.4)	67.4 (49.9, 94.8)	0.45	48.3 (34.8, 66.0)	50.5 (34.7, 70.1)	0.50
***GFR ml/min/1.73*** ***m^2^***	91.3±23.0	84.2±19.3	<0.0001	93.1±20.2	87.2±19.1	0.003
***Calcium (mmol/L)***	2.27±0.09	2.28±0.09	0.30	2.26±1.11	2.27±0.10	0.80
***Cholesterol (mmol/L)***	4.02±0.95	4.48±1.05	0.001	4.86±1.03	5.11±1.17	0.04
***BMI (kg/m^2^)***	24.0 (20.0, 29.0)	30.9 (27.4, 35.1)	<0.0001	24.9 (21.1, 29.6)	28.8 (25.3, 32.6)	<0.0001

Data given as mean ±SD for normally distributed data and median (IQR) for non-normally distributed data.

In a univariate analysis there was a significant negative correlation between PTH and 25(OH) D (p<0.0001, see Table 4) and a significant trend for PTH to be higher in Indian subjects (p = 0.04) and females (p = 0.0002). We also observed significant (p<0.05) positive associations of PTH with age, BMI, glucose, HOMA, triglyceride, cholesterol, waist circumference and systolic and diastolic blood pressures. The 25(OH)D levels were correlated negatively with BMI, and GFR and correlated positively with systolic and diastolic blood pressure (p<0.05 for all associations). There were significant trends for 25(OH)D to be higher in African and male subjects (p<0.001 for all comparisons).

**Table 4:** Pearson’s correlations for PTH and 25(OH)D for all subjects.

Variable	Log PTH	Log 25(OH)D
***Age***	0.26 (<0.0001)	−0.02(0.52)
***Log BMI***	0.22 (0.0001)	−0.03 (0.38)
***GFR***	−0.07 (0.05)	−0.11 (0.003)
***Log Glucose***	0.09 (0.02)	−0.06 (0.09)
***Cholesterol***	0.11 (0.005)	−0.17 (<0.0001)
***Log Triglyceride***	0.14 (<0.0001)	−0.08 (0.02)
***HDL-C***	0.009 (0.81)	−0.06 (0.08)
***Log Systolic blood pressure***	0.15 (0.0001)	0.12 (0.001)
***Log Diastolic blood pressure***	0.19 (0.0001)	0.08 (0.02)
***Log HOMA***	0.09 (0.02)	−0.02 (0.67)
***Log PTH***	–	−0.19 (<0.0001)
***Log 25(OH)D***	−0.24 (<0.0001)	–
***Log Waist***	0.25 (<0.0001)	0.0002 (0.99)

Data given as r (p-value).

Multivariate associations of 25(OH)D and PTH with components of the Met S are presented in table 5, with age, gender and BMI included as independent variables in all models. Addition of ethnicity to all the models demonstrated that Indian ethnicity was a positive determinant of glucose, triglyceride and HOMA levels but a negative determinant of diastolic and systolic blood pressure. Addition of 25(OH)D to these models had minimal effect on the beta values for ethnicity and in none of the models was 25(OH)D shown to be a determinant of any of the components of the metabolic syndrome, or HOMA. When PTH was added to each of the models, neither the ethnicity or 25(OH)D beta values were affected, however PTH was shown to be positively and significantly associated with systolic (p = 0.018) and diastolic (p = 0.005) blood pressures and waist circumference (p<0.0001) and negatively associated with HOMA (p = 0.008) levels.

**Table 5:** Multiple regression models for determining the effects of ethnicity, PTH and 25(OH)D on components of the metabolic syndrome.

Dependent variable	Model numbers and independent variables					
	MODEL 1	MODEL 2		MODEL 3		
	Ethnicity	Ethnicity	25(OH)D (log)	Ethnicity	25(OH)D (log)	PTH (log)
***Log Glucose***	0.02 (0.013)	0.02 (0.015)	−0.003 (0.47)	0.02 (0.015)	−0.003 (0.44)	−0.01 (0.43)
***Log Triglycerides***	0.13 (<0.0001)	0.13 (<0.0001)	−0.001 (0.89)	0.13 (<0.0001)	0.0007 (0.94)	0.04 (0.32)
***Log HDL-C***	0.0002 (0.99)	−0.0007 (0.93)	−0.007 (0.11)	−0.0009 (0.93)	−0.006 (0.175)	−0.005 (0.78)
***Log Systolic blood pressure***	−0.04 (<0.0001)	−0.04 (<0.0001)	0.002 (0.34)	−0.04 (<0.0001)	0.002 (0.28)	0.02 (0.018)
***Log Diastolic blood pressure***	−0.02 (<0.0001)	−0.02 (<0.0001)	0.003 (0.14)	−0.02 (<0.0001)	0.004 (0.11)	0.03 (0.005)
***Log Waist***	0.02 (0.002)	0.02 (0.001)	0.003 (0.37)	0.02 (0.005)	0.004 (0.19)	0.09 (<0.0001)
***Log HOMA***	0.23 (<0.0001)	0.23 (<0.0001)	0.0009 (0.93)	0.23 (<0.0001)	−0.0003 (0.97)	−0.13 (0.008)

Data given as beta (p-value); all models adjusted for age, gender and BMI. Ethnicity, 25(OH) D and PTH were added in a forward, stepwise manner.

Logistic regression was used to determine whether the higher prevalence of Met S in the Indian population was related to PTH or 25(OH)D levels. The data in Table 6 shows that after adjusting for age, gender and BMI Asian Indian ethnicity carries a 2.24 (95% CIs 1.57, 3.18; p<0.0001) increased risk of Met S compared to African subjects (model 1). Additional adjustment for 25(OH)D had no effect on the p-value (or odds ratio (OR)) for ethnicity and had no significant effect itself (model 2). The final addition of PTH to the logistic regression model (model 3) had no effect on the p-value or OR for ethnicity or 25(OH)D but did have a significant effect itself (OR = 2.48; 95% CIs 1.01, 6.08; p = 0.04).

**Table 6:** Multivariate logistic regression analysis for determining the effect of ethnicity, PTH and 25(OH)D on risk of metabolic syndrome.

Modelnumbers	Independent variables	Odds ratios	95% CIs	p-values
*1*	Ethnicity	2.24	1.57, 3.18	<0.0001
*2*	Ethnicity	2.29	1.60, 3.28	<0.0001
	Log 25(OH)D	1.15	0.78, 1.70	0.47
*3*	Ethnicity	2.27	1.56, 3.30	<0.0001
	Log 25(OH)D	1.25	0.67, 2.24	0.48
	Log PTH	2.48	1.01, 6.08	0.04

All models adjusted for age, gender and BMI, with metabolic syndrome as the dependent variable. Ethnicity, 25(OH)D and PTH were added in a forward, stepwise manner.

In order to determine which individual components of the Met S are influenced by PTH to increase the risk of Met S, logistic regression analysis was performed. The results in Table 7 show that the increased risk for Met S conferred by PTH arises principally through its effect on systolic blood pressure and its positive association with waist circumference. This is demonstrated by the fact that adding these variables individually to the logistic regression model attenuates the p-value of the OR for PTH (see Table 7, models 6 and 7). Adding both these variables to the regression model at the same time attenuates the p-value for the PTH OR to a greater extent than adding each variable on its own (see Table 7, model 8). This suggests that the effects of each variable are additive and independent. Addition of the other metabolic syndrome components to the logistic regression model does not attenuate the p-value/OR for PTH and in some cases increases it. The exception is glucose, which does have a weak effect on the p-value for PTH, increasing it from 0.04 to 0.09 (see Table 7, model 4).

**Table 7:** Risk of metabolic syndrome due to PTH and the effects of adjusting for individual components of the metabolic syndrome on PTH odds ratios in a logistic regression analysis.

Model number	Independent variables	Odds ratios	95% CIs	p-values
***1***	Log PTH	2.48	1.01, 6.08	0.04
***2***	Log PTH	2.91	1.08, 7.83	0.03
	T riglyceride ≥1.7 mmol/L	11.9	7.19, 19.6	<0.0001
***3***	Log PTH	3.08	1.09, 8.76	0.03
	HDL-C <1.3 mmol/l (females) or <1.0 mmol/L (males)	13.3	8.41, 21.0	<0.0001
***4***	Log PTH	2.28	0.87, 5.97	0.09
	Glucose ≥5.6 mmol/L	9.58	5.90, 15.6	<0.0001
***5***	Log PTH	2.76	1.05, 7.24	0.04
	Diastolic bp≥85 mm/Hg	5.85	3.92, 8.73	<0.0001
***6***	Log PTH	1.76	0.66, 4.65	0.25
	Systolic bp≥130 mm/Hg	8.32	5.28, 13.1	<0.0001
***7***	Log PTH	2.03	0.82, 5.06	0.13
	Waist ≥94 or ≥90 cm for African or Indian males or ≥80 cm for females	9.69	4.55, 20.6	<0.0001
***8***	Log PTH	1.43	0.53, 3.85	0.48
	Systolic bp≥130 mm/Hg	8.46	5.32, 13.5	<0.0001
	Waist ≥94 or ≥90 cm for African or Indian males or ≥80 cm for females	10.3	4.66, 22.7	<0.0001

All models adjusted for age, gender, ethnicity, BMI and 25(OH)D with metabolic syndrome as dependent variable.

## Discussion

In this study we investigated whether 25(OH)D or PTH is associated with the Met S or its components. Our results show a significant association of PTH, but not 25(OH)D, with the Met S. Higher PTH levels were associated with increased systolic and diastolic blood pressure and reduced insulin resistance as assessed by the HOMA index and an increased odds ratio for the Met S. Furthermore our results suggest that neither 25(OH)D nor PTH contribute to ethnic differences in the prevalence of Met S between African and Asian Indian subjects. There are no previous studies from sub-Saharan Africa that have examined the relationship between 25(OH)D, PTH and the Met S. Our results also showed lower levels of 25(OH)D in Asian Indians compared to Africans. Studies from India have also shown that this population tends to have poor vitamin D status [22], [23].

Our observations of a lack of association of 25(OH)D with Met S are in line with those of Scragg et al. who observed no association between 25(OH)D status and type 2 diabetes in non-Hispanic black subjects but did observe a negative associations between 25(OH)D and risk of diabetes in Mexican Americans and non-Hispanic white populations [24]. Similarly, in a single study from India, Majumdar et al. showed that although 25(OH)D insufficiency was highly prevalent it was not associated with the Met S or insulin resistance [25].

The results of studies on the association of 25(OH)D and PTH with the Met S, components of the Met S or related disorders have been inconsistent. In a population based cross sectional study of US men and women over 20 years-of-age (NHANES III,1988–94) the prevalence of Met S and its components fell across increasing quartiles of 25(OH)D after adjustment for age, race, sex, income, lifestyle factors, calcium and energy intake. This study also showed that the odds ratio for Met S increased with increasing PTH in older men only [26]. In a study carried out in an aging European population, investigators showed that the risk for Met S decreased across increasing quintiles of 25(OH)D but could show no association of PTH with Met S [10]. Neither of these studies adjusted for BMI. However, Reis et al. [27] showed a lack of association between serum 25(OH)D and Met S in an adult Caucasian population. Our population, particularly the African subset, was not particularly vitamin D deficient overall (median 25(OH)D in Africans was 71 nmol/l) and 25(OH) D may be more strongly associated with Met S in a deficient population. However, this cannot explain the lack of association of 25(OH)D with Met S in the Asian Indian group, as serum 25(OH)D levels were significantly lower in this population compared to the African cohort.

A number of other studies have implicated PTH as being associated with the Met S rather than 25(OH)D [10], [11]. Several lines of evidence support a role of PTH in increasing the risk of CVD. Thus, it has been associated with increased cardiovascular mortality in selected population groups [28], [29], [30] and with increased coronary heart disease in a younger group [31]. This increased risk may be mediated via its effects on blood pressure, insulin resistance, hyperglycaemia and low HDL-C [26], [27]. In multivariate regression models we confirmed a positive relationship between PTH and blood pressure but could not verify any relationship between PTH and blood glucose or lipid levels, although significant associations were observed in univariate analyses. The negative relationship that we observed between PTH and the HOMA index was only observed after adjusting for confounding variables within a multivariate regression analysis. In a univariate analysis PTH correlated positively with HOMA. Derangements in glucose metabolism in primary hyperparathyroidism are well described with increased PTH increasing glucose intolerance [26], [32], [33], [34]. A few studies have described a positive relationship between PTH and insulin resistance similar to our observation in univariate analyses. Some investigators have postulated that this relationship may be secondary to hypercalcaemia as it is absent in normocalcaemic subjects [35], [36]. Frost et al. showed a significant negative association of PTH with insulin resistance in young men [37]. The negative relationship we have noted between PTH and HOMA in a multivariate analysis may be due to a number of reasons: ethnic differences, the fact that our subjects were all normocalcaemic or the adjustment for confounders. In addition to its possible effect on insulin sensitivity clinical evidence also supports a role of PTH in increasing blood pressure [13] and observational studies have linked elevated PTH levels to an increased risk of hypertension, left ventricular hypertrophy, and cardiovascular morbidity and mortality [30]. It is thought that PTH mediates its effect by directly increasing the secretion of aldosterone from the adrenal glands and indirectly by activating the renin-angiotensin system [38].

Logistic regression models demonstrated that the increased risk of Met S in subjects with higher PTH levels is largely due to the positive and independent relationships of systolic blood pressure and waist circumference with PTH. Previous studies have also shown a positive association of PTH with obesity [30], [39]. Furthermore body weight changes correlate positively with changes in serum PTH levels [40], [41] suggesting that obesity may be causative for hyperparathyroidism. There are no studies in the literature to show that PTH is secreted by adipocytes but there are studies that demonstrate that PTH does modify adipocyte function. These effects include the inhibition of adipocyte lipoprotein lipase activity [42] and the attenuation of insulin signalling [43]. However, both these actions of PTH would limit rather than augment triglyceride deposition within adipose tissue. It is possible that increased adiposity may lead to greater production of PTH by the parathyroid, and it is interesting to note that studies have shown a positive correlation between serum leptin and PTH concentrations [44], [45].

Our data show that Asian Indian ethnicity is associated with increased risk for Met S and most of its components. As a group, Asian Indians tend to be insulin resistant and at high risk for diabetes and premature coronary heart disease when compared to other ethnic groups [46] and this is partly explained by high visceral fat content [47]. In the present study ethnic differences in the levels of Met S-related metabolic variables and the prevalence of Met S were investigated using multiple regression and logistic regression analyses and were found not to be related to ethnic differences in PTH or 25(OH)D levels.

This study is limited by the cross-sectional nature of the data, and as such we cannot draw any conclusions about causality. Another limitation is that blood samples were collected over several months, and vitamin D is known to display seasonal variation. Also, study subjects were not selected randomly and this may have introduced some level of selection bias. Finally, 25(OH)D levels were not measured using the reference method of liquid chromatography-tandem mass spectrometry (LC-MS/MS), however the technique used in this study (HPLC) has been shown to correlate well with the LC-MS/MS method [48].

In conclusion, PTH but not 25(OH)D is associated with the Met S in our African and Asian Indian populations. The PTH effect is largely via its impact on blood pressure and its relationship with waist circumference. The relationship between PTH and insulin resistance needs to be investigated further, as does the mechanism responsible for causing serum PTH levels to rise with increased adiposity.
